# Myeloid-Derived Suppressor Cells in Cancer: Metabolic Reprogramming, Immune Crosstalk, and Therapeutic Targeting

**DOI:** 10.3390/cancers18132150

**Published:** 2026-07-03

**Authors:** Andrea Sabatini, Maria Rita Assenza, Maria Teresa Bilotta, Paola Vacca, Nicola Tumino

**Affiliations:** Innate Lymphoid Cells Unit, Bambino Gesù Children’s Hospital, IRCCS, 00146 Rome, Italy; andrea.sabatini@opbg.net (A.S.); mariarita.assenza@opbg.net (M.R.A.); nicola.tumino@opbg.net (N.T.)

**Keywords:** myeloid-derived suppressor cells, TME, immune crosstalk, cancer, metabolism, cancer therapy

## Abstract

Myeloid-derived suppressor cells (MDSCs) are a heterogeneous population of immature myeloid cells that accumulate in cancer and represent one of the major drivers of tumor-associated immunosuppression. MDSCs actively contribute to tumor progression by inhibiting both innate and adaptive immune responses, promoting angiogenesis, metastatic dissemination, and resistance to immunotherapy. Two major subsets have been identified, polymorphonuclear (PMN-) and monocytic (Mo-) MDSCs, each characterized by distinct phenotypic, metabolic, and suppressive properties. Within the tumor microenvironment (TME), MDSCs establish a complex network of interactions with T-, B-, NK-cells, dendritic cells, and macrophages, thereby orchestrating immune escape and tumor persistence. Recent evidence highlights the pivotal role of metabolic rewiring in regulating MDSC survival and suppressive activity. Enhanced aerobic glycolysis, fatty acid oxidation, amino acid depletion, reactive oxygen species (ROS) production, and adenosine metabolism collectively sustain MDSC-mediated immune dysfunction and shape the immunosuppressive TME. In particular, the crosstalk between PMN-MDSCs and NK cells has emerged as a critical mechanism of tumor immune evasion, leading to impaired NK cell cytotoxicity, altered activating receptor expression, and defective cytokine production. In this review, we summarize the current knowledge on the phenotypic and functional heterogeneity of MDSCs, their metabolic adaptations, and their interactions with immune effector populations in cancer. Furthermore, we discuss emerging therapeutic strategies aimed at targeting MDSC recruitment, differentiation, metabolic pathways, and suppressive functions. Understanding the molecular and metabolic mechanisms governing MDSC biology may provide novel opportunities to overcome tumor-induced immunosuppression and improve the efficacy of current cancer immunotherapies.

## Simple Summary

Myeloid-derived suppressor cells are a group of immature immune cells that accumulate in various types of cancer. They hinder the immune system’s ability to recognize and eliminate tumor cells. By compromising the body’s natural defenses, these cells facilitate tumor growth and spread and reduce responsiveness to treatments, including immunotherapy. In this review, we summarize current knowledge about the development of myeloid-derived suppressor cells, their interactions with other immune cells, and their adaptation of energy use to survive within the tumor microenvironment. These adaptations enable them to maintain their potent immune-suppressing activity and further promote cancer progression. We also discuss new therapeutic strategies aimed at preventing their recruitment to tumors, reducing their numbers, and interfering with the biological processes that support their function. Although many of these approaches are still being researched, targeting myeloid-derived suppressor cells is a promising strategy to restore effective anti-tumor immunity and enhance the success of existing cancer treatments. A better understanding of these cells could lead to the development of more effective and personalized therapies for cancer patients.

## 1. Introduction

Myeloid-derived suppressor cells (MDSCs) are a heterogeneous subpopulation with a potent regulatory role that modulates immune responses in the inflammatory milieu. The immunosuppressive role of MDSCs may be dual: beneficial in contexts such as pregnancy, sepsis, trauma, and inflammatory diseases [1,2,3], or detrimental in contexts such as infections or cancer [1].

MDSCs are virtually absent under physiological conditions but are abundant in both peripheral blood (PB) and tissues during pathological states, including hematological [2,3] and solid tumors [4,5]. The first studies on the accumulation of myeloid cells in cancer were published in the late ’70s [6], but subsequent investigations demonstrated the existence of distinct myeloid subsets with immunosuppressive functions [5,6], although many aspects of their complex biology remain to be fully elucidated. MDSCs contribute to tumor progression through tumor angiogenesis [7], drug resistance [8], and promotion of tumor metastases [9], limiting the efficacy of immunotherapy in cancer [10,11]. In the tumor microenvironment (TME), MDSCs can affect the capability of the immune response to eliminate tumor cells and help them to evade the immune response, spread, and form metastasis through different mechanisms [12,13]. Understanding the immunosuppressive mechanisms, the interaction with other immune cells, and therefore strategies to target these cells may represent an important milestone for cancer treatments [8].

In this review, we discuss the phenotypic and functional heterogeneity of MDSCs as well as the suppressive mechanisms, the interactions with immune cells (e.g., T-cells, B-cells, and natural killer cells), the metabolic reprogramming, and therapeutic strategies, aiding in overcoming resistance to cancer immunotherapies. Unlike previous reviews, we offer an integrated and translational perspective that connects the metabolic underpinnings of MDSC suppressive activity to immune cell crosstalk and to actionable therapeutic targeting strategies, providing a more cohesive framework for understanding MDSC-driven immune evasion in cancer.

## 2. Phenotypic and Functional Characterization of MDSCs

MDSCs are a heterogeneous population with diverse morphological, phenotypic, and functional features. Consistent with this, they express a range of myeloid-associated surface markers while lacking lineage-specific markers of B- and T-lymphocytes, natural killer (NK) cells, macrophages, and dendritic cells (DCs). MDSCs consist of two major subsets of described cells, polymorphonuclear (PMN-) and monocytic (Mo-) MDSCs. Morphologically, PMN-MDSCs are similar to neutrophils, while Mo-MDSCs are similar to monocytes. These cells have been phenotypically characterized by flow cytometry, and the criteria for the nomenclature and characterization standards have been defined [14]. In mice, MDSCs co-express CD11b and Gr-1 and are classified into PMN-MDSCs (Ly6G^+^Ly6C^lo^) and Mo-MDSCs (Ly6G^−^Ly6C^hi^) [15,16]. In humans, MDSCs are identified as Lineage (CD3-14-19-56-123)^−^, CD11b^+^, CD33^+^, HLA-DR^low/−^, and subdivided into two subsets: Mo-MDSCs are CD14^+^CD15^−^ cells, and PMN-MDSCs are CD14^−^CD15^+^CD66b^+^CD124^+^ [17]. 

Until recently, separation of neutrophils from PMN-MDSCs relied on density gradient centrifugation, whereby PMN-MDSCs are enriched in the low-density peripheral blood mononuclear cells (PBMC) fraction, whereas neutrophils segregate with high-density cells [1]. Additional markers, such as lectin-type oxidized LDL receptor 1 (LOX-1) and S100A9, have emerged as candidate markers of human PMN-MDSCs [18].

MDSCs share a common myeloid progenitor (CMP) with other myeloid cells (e.g., macrophages, granulocytes, and DCs). MDSC development is governed by the same growth factors that control normal myelopoiesis, e.g., macrophage colony-stimulating factor (M-CSF), granulocyte colony-stimulating factor (G-CSF), and granulocyte-monocyte colony-stimulating factor (GM-CSF). These mediators, together with IL-6, are abundant in pathological conditions and TME [19]. In particular, tumor-derived GM-CSF plays a key role in the generation, maintenance, and survival of MDSCs, and its sustained production may underlie the marked accumulation of these cells observed in cancer patients [11,14]. GM-CSF, together with IL-6, can activate the JAK/STAT pathway, involving STAT3 and STAT5, which induces the expression of key genes that drive the proliferation, survival, and suppressive activity of MDSCs [20]. Elevated serum levels of these cytokines have been associated with increased frequencies of circulating MDSCs.

## 3. Immunosuppressive Mechanisms of MDSCs

**Figure 1:**
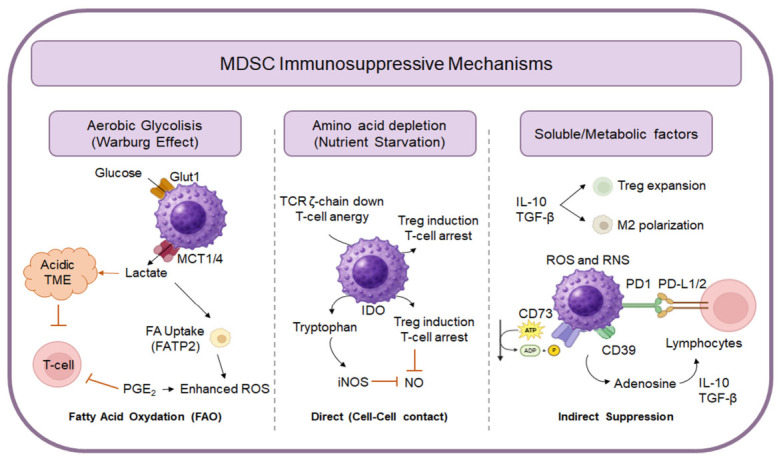
Schematic representation of the major immunosuppressive mechanisms exerted by MDSCs. MDSCs suppress anti-tumor immunity through multiple interconnected mechanisms encompassing metabolic reprogramming, amino acid depletion, and intercellular signaling. Aerobic glycolysis and fatty acid oxidation alter the TME and generate immunosuppressive mediators, including lactate, ROS, and PGE2. Depletion of key amino acids such as ʟ-arginine and tryptophan, mediated by Arg-1, IDO, and iNOS, impairs T-cell activation and promotes Treg induction. Indirect suppression is further sustained by secretion of IL-10 and TGF-β and by purinergic adenosine signaling. Direct cell-contact suppression occurs through upregulation of immune checkpoints, including PD-L1, driving T-cell exhaustion.

The main characteristic of MDSCs is the ability to suppress immune effector cells, including those from innate (monocytes, DCs, NK cells) and adaptive (T- and B-cells) compartments (Figure 1).

They can produce different cytokines and soluble factors that have direct immunoregulatory effects, such as IL-10, TGF-β, reactive oxygen (ROS) and nitrogen species (RNS), nitric oxide (NO), cyclooxygenase 2 (COX-2), and indoleamine 2,3-dioxygenase (IDO), or arginase (Arg) 1 and 2 [8]. Moreover, immune regulation is enhanced by the contextual recruitment of other regulatory cells, including regulatory T (Treg) and B cells (Breg), M2 macrophages, and tolerogenic DCs (TolDCs) [21]. MDSCs rewire their metabolic pathways, specifically involving glucose, lipids, amino acids, and adenosine, to survive in hostile microenvironments and effectively suppress immune responses.

One of the key features of MDSCs is the nutrient depletion induced by the production of enzymes like Arg-1 and -2, IDO, and iNOS. These molecules deplete arginine (ʟ-Arg), cysteine, and tryptophan, three important amino acids involved in lymphocyte proliferation and function, leading to a progressive immune cell anergy [22]. Arg-1 degrades ʟ-Arg, which is vital for T-cell receptor signaling, while the enzyme IDO breaks down tryptophan into kynurenine, a metabolite that inhibits T-cell proliferation and promotes the development of Tregs [23,24,25,26,27,28].

As already mentioned, another MDSC feature is reactive species production. Increased ROS and RNS secretion disturb the equilibrium status of pro-oxidant/antioxidant reactions and is also important for the impairment of immune cells, especially T-cells. Indeed, ROS catalyzes the nitration of TCR/CD8 molecules to prevent the TCR/MHC–peptide interactions [29,30].

Secretion of IL-10 and TGF-β is pivotal in shifting responses toward Tregs and suppressive macrophages, ensuring the formation of an immunosuppressive environment [31].

MDSCs are also involved in cell–cell communication, upregulating immune checkpoints, and driving T-cell exhaustion. In this context, an important role is played by Programmed Death-Ligand 1 (PD-L1), which is expressed by MDSCs and inhibits cell proliferation, survival, cytokine production, and other effector functions upon interaction with PD-1. Besides adaptive immune cells, MDSCs can incite NK and DC impairment through a decrease in NK cytotoxicity, DC maturation, and antigen presentation [32].

MDSCs heavily utilize aerobic glycolysis (the Warburg effect), converting glucose into lactate even when oxygen is present. This high glucose consumption depletes resources for immune cells, while the resulting lactate accumulation acidifies the environment and impairs cell function by disrupting their redox balance [33,34,35]. MDSCs also rely on fatty acid oxidation (FAO) to generate energy and ROS, which enhances their suppressive activity. Tumor-derived factors like GM-CSF induce the transporter fatty acid transport protein 2 (FATP2), allowing MDSCs to take up arachidonic acid and produce PGE2, a major mediator of immune suppression [36,37,38].

Finally, in the TME, MDSCs use the enzymes CD39 and CD73 to convert extracellular ATP into adenosine that binds their receptors (e.g., A2AR/A2BR) on MDSCs, triggering signaling pathways that increase the production of immunosuppressive molecules like TGF-β and IL-10 [39,40,41,42,43].

## 4. MDSCs and Immune Cell Interactions in Cancer

**Figure 2:**
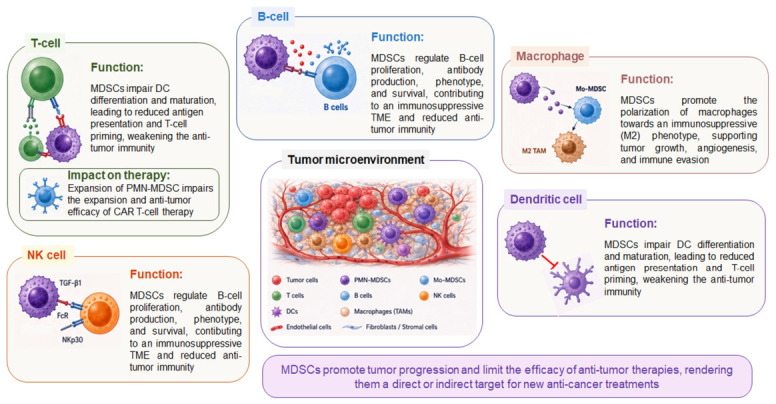
Interactions between MDSCs and immune cells within the TME. The TME is a complex and heterogeneous ecosystem composed of malignant cells and multiple stromal and immune cell populations. Within the TME, MDSCs play a central role in tumor-induced immunosuppression by releasing soluble mediators and engaging multiple molecular pathways that impair the function of T-cells, B-cells, NK-cells, DCs, and macrophages, thereby promoting immune evasion and tumor progression.

The TME is a heterogeneous ecosystem in which malignant cells coexist with non-tumoral elements, including mesenchymal stromal cells, endothelial cells, fibroblasts, and a spectrum of immune cell populations. Within the TME, PMN-MDSCs constitute the predominant subset of MDSCs, accounting for about 80% of the total MDSC population. MDSCs play a central role in orchestrating immunosuppression within the TME, employing a broad array of mediators and molecular mechanisms to inhibit several critical immune effectors, including T-, B-, NK-ells, DC, and macrophages [44,45] (Figure 2).

### 4.1. T-Cells

MDSCs are potent inhibitors of T-cell immune responses [46] and employ multiple mechanisms to suppress T-cell function. As mentioned before, one major pathway involves the modulation of amino acid metabolism. In in vivo models, MDSCs impair T-cell activation by sequestering cystine and limiting its availability. Because T-cells are unable to efficiently synthesize or import cysteine, they rely on antigen-presenting cells (APCs), such as macrophages and DCs, for their supply. In contrast, MDSCs take up cystine but do not release cysteine, thereby competing with APCs and reducing extracellular cysteine levels, ultimately impairing T-cell activation [47].

Arg-1 and Arg-2 enzymes reduce ʟ-Arg availability, impairing T-cell activation and proliferation by downregulating the CD3ζ chain of the TCR complex [48]. The accumulation of Arg-1^+^ MDSCs results in systemic ʟ-Arg depletion, which is associated with tumor progression [49]. Notably, T-cell response suppression can be reversed by arginase inhibition or ʟ-Arg supplementation [50]. In PBMC and tissue samples from lung cancer patients, increased frequencies of PMN-MDSCs correlate with reduced CD4^+^ and CD8^+^ T-cell populations, and elevated Arg-1 mRNA expression in PBMC is associated with MDSC abundance and suppressive activity [51,52]. Similar Arg-1 upregulation has been reported across multiple tumor types, including gastric [53,54], colorectal [55], renal [56], and hepatocellular carcinomas [57].

In melanoma and colorectal cancer patients, interactions between MDSCs and activated T-cells induce IL-10 production, which promotes STAT3 activation in MDSCs, leading to an upregulation of PD-L1 expression. These PD-L1^+^ MDSCs mediate immunosuppression through mechanisms involving Arg-1 and IDO [58]. IDO overexpression by MDSCs leads to tryptophan depletion, resulting in T-cell cycle arrest and impaired immune function [59]. PMN-MDSCs also release ROS into the extracellular space [60], thereby directly and indirectly promoting tumor progression. In tumor mouse models, ROS production by PMN-MDSCs suppresses T-cell responses through pathways involving FATP2 and STAT3 signaling [61].

New treatment approaches based on adoptive cell therapy (CAR T-cells) could be influenced by the presence and expansion of PMN-MDSCs in the PB of hematological and solid tumor-treated patients. The expansion of PMN-MDSCs affects the expansion and the potent anti-tumor activity of CAR T-cells [11].

### 4.2. B-Cells

B-cells contribute to tumor progression through multiple mechanisms, including the production of lymphotoxin, which induces angiogenesis and tumor growth. Tumor-derived extracellular vesicles can activate B-cells to produce antibodies that form immune complexes, activating Fcγ receptors on myeloid cells, and driving their differentiation into MDSCs [62]. Despite extensive research, evidence supporting crosstalk between MDSCs and B-cells remains limited. In a cancer mouse model, MDSCs were found to accumulate around splenic germinal centers, co-localizing with B-cells and enhancing their proliferation and IgA-production, mediated by IL-10 and TGF-β1 [63]. A similar mechanism involving direct cell-to-cell contact has also been described in a lung cancer model, where MDSCs suppress B-cell proliferation in an Arg-dependent manner [64]. In tumor-bearing mice, a reduction in B-cells in the bone marrow was linked to the infiltration of MDSCs. Tumor progression disrupted key pathways for B-cell development, including IL-7 and STAT5 signaling, leading to decreased serum IgG levels. The extent of B-cell impairment correlated positively with MDSC infiltration and cancer prognosis [64]. In breast cancer mouse models, MDSCs and splenic B-cells increased significantly. MDSCs altered B-cell phenotypes, affecting immune checkpoints like PD-1 and PD-L1, and regulating functions such as proliferation, apoptosis, antibody production, and cytokine secretion [65].

MDSCs, Tregs, and Bregs play a significant role in the immunosuppressive microenvironment of glioblastoma (GBM) in both preclinical models and patient samples. Bregs exhibited immunosuppressive activity against activated CD8^+^ T-cells and overexpressed inhibitory molecules like PD-L1 and CD155, along with producing TGF-β and IL-10. Additionally, MDSCs enhance the immunosuppressive effects of Bregs by transferring PD-L1 via microvesicles, creating a critical MDSC–B-cell axis that contributes to immune suppression in GBM [21].

Studies in human patients remain relatively limited. Bodogai et al., showed that MDSCs from B-cell chronic lymphocytic leukemia patients displayed only partial regulatory function unless educated by Bregs via TGF-β receptor signaling. This interaction enhanced MDSC production of ROS and NO, strengthening their ability to suppress CD4^+^ and CD8^+^ T-cells, and promoting tumor growth and metastasis. Disruption of Bregs or TGF-β signaling impaired MDSC function and blocked metastasis [66]. PMN-MDSCs can also differentially regulate B-cell function by suppressing their proliferation and antibody production in a cell contact-dependent manner. This effect involves established mediators (i.e., Arg-1, NO, ROS) and is associated with the induction of B-cell death [67].

A study found that human Mo-MDSCs inhibit B-cell proliferation and function in vitro through soluble mediators like IDO, Arg-1, and NO, without direct contact. They also alter B-cell phenotype by downregulating activation markers and affecting gene expression related to apoptosis, class-switch regulation, and B-cell differentiation [68].

### 4.3. NK Cells

NK cells are key effectors of antitumor immunity, primarily acting through their cytotoxic capacity and the secretion of soluble factors and cytokines, which can directly eliminate tumor cells or indirectly shape the immune response by recruiting and activating other immune populations [69].

Several experimental models have demonstrated that MDSCs exert a potent inhibitory effect on NK cell function. In multiple murine tumor models, NK cell activity is significantly impaired, a phenomenon closely associated with MDSC accumulation. This dysfunction is characterized by reduced cytotoxicity, decreased expression of activating receptors, and impaired IFN-γ production. Mechanistically, MDSCs inhibit NK cell activation predominantly through membrane-bound TGF-β1 [70,71]. Consequentially, NK cell activation can counteract MDSC-mediated immunosuppression by reducing their accumulation via the NKG2D-NKG2DL axis and promoting their maturation through IFN-γ-dependent signaling, an effect associated with improved clinical outcomes [72]. Additional suppressive mechanisms involve NO production and activation of the IL-6/STAT3 axis, both of which contribute to NK cell dysfunction and tumor immune escape [73,74]. Among the inhibitory mechanisms, as mentioned above, one involves direct cell-to-cell contact. In vitro co-culture systems have shown that MDSCs suppress NK cell functions, including antibody-dependent cellular cytotoxicity, cytokine production, and downstream signaling, through Fc receptors on NK cells [73]. Additional studies in hepatocarcinoma and lung cancer further demonstrate that MDSCs inhibit NK cell activity via receptors such as NKp30 [4,75].

PMN-MDSCs are highly enriched in the PB and TME of lung cancer patients, where their accumulation correlates with poor clinical outcome and reduced NK cell frequency and functionality. PMN-MDSCs profoundly impair NK cell activity by downregulating activating receptors, reducing cytotoxicity, degranulation, and cytokine production through both cell-to-cell contact and soluble mediators, including PMN-MDSC-derived exosomes. Importantly, these findings identify PMN-MDSCs as key regulators of NK cell dysfunction and suggest that targeting PMN-MDSC-mediated immunosuppressive mechanisms may represent a promising strategy to restore NK cell-mediated anti-tumor immunity [4].

In clinical contexts, MDSC-mediated suppression is also evident in hematological malignancies. In myelodysplastic syndromes, co-culture with MDSCs results in reduced expression of NK activating receptors (NKG2D, NKp30, NKp46), decreased effector molecule production (CD107a, IFN-γ, perforin, and granzyme B), and increased apoptosis of NK cells. Importantly, blockade of the TIGIT/CD155 axis partially restores NK cell function, highlighting an additional checkpoint involved in MDSC-mediated immune suppression [76].

Finally, clinical studies support the immunoregulatory role of MDSCs. In patients with head and neck squamous cell carcinoma (HNSCC), elevated levels of circulating and tumor-infiltrating PMN-MDSCs and Mo-MDSCs correlate with strong immunosuppressive activity, driven by TGF-β and NO pathways [77]. In the context of immunotherapy, a higher NK cell-to-Lox-1^+^ PMN-MDSC ratio has been associated with better responses to anti-PD-1 therapy, longer progression-free survival, and improved overall survival [78]. A small-molecule screen identified PI3K-γ inhibitors as effective modulators of MDSCs’ function [79]. Finally, in sarcoma and breast cancer patients, IL-6 produced by tumor-infiltrating NK cells correlates with MDSC-associated markers, such as S100A8/9 and Arg-1, further linking NK-MDSC crosstalk to tumor progression and immune remodeling [74].

### 4.4. Dendritic Cells

DCs are specialized APCs that connect innate and adaptive immunity by providing signals essential for T-cell activation and differentiation. Tumor-related conditions lead to the accumulation of immature DCs and hinder their maturation, causing functional impairments [80,81,82].

The accumulation of MDSCs represents a key mechanism underlying defective DC differentiation in cancer, thereby contributing to defective antitumor immunity. In murine models, this process is mediated in part by the STAT3/S100A9 axis, which promotes MDSC expansion and inhibits DC development [83]. Mechanistically, PMN-MDSCs selectively impair DC cross-presentation, without affecting direct antigen presentation, via a contact-independent mechanism involving the transfer of oxidized lipids. Although both neutrophils and PMN-MDSCs transfer lipids to DCs, only PMN-MDSCs suppress cross-presentation due to their capacity to generate oxidatively truncated lipids, driven by myeloperoxidase (MPO) [84].

Consistent with these findings, in vitro studies have shown that mouse MDSCs differentiated from c-kit^+^ bone marrow progenitor cells in the presence of IL-4, GM-CSF, and PGE2 are associated with a proportional decline in the number of mature DCs [85]. Similarly, stimulation with LPS and IFN-γ inhibits DC development while enhancing MDSC suppressive functions [86]. In several cancer types, patients exhibit decreased DC levels alongside increased MDSC frequencies, correlating with poorer overall and progression-free survival [87]. Finally, patients with HNSCC display elevated levels of Mo-MDSCs and tolerogenic DCs, both of which suppress activated T-cells through distinct signaling pathways. PI3K-AKT signaling is critical for the induction and activity of Mo-MDSCs, whereas β-catenin-dependent Wnt signaling drives the tolerogenic properties of DCs [88].

### 4.5. Macrophages

Macrophages support host defense by regulating adaptive immunity, promoting tissue repair, and eliminating pathogens. In cancer, however, they represent a highly heterogeneous population: M1-macrophages promote anti-tumor immunity, while M2-macrophages exhibit immunosuppressive functions and support tumor growth [89]. Within the TME, tumor-associated macrophages (TAMs) are predominantly polarized towards an M2 phenotype, which contributes to tumor progression by promoting angiogenesis, tumor cell invasion, and metastasis [81]. The immunosuppressive activity of TAMs is further reinforced by MDSCs. These interactions modulate key mediators, such as IL-6, IL-10, TNF-α, and NO. For instance, MDSC-derived IL-10 suppresses macrophage pro-inflammatory cytokines while enhancing NO production [81]. In parallel, PMN-MDSCs can promote the differentiation of Mo-MDSCs into M2-macrophages via exosome-mediated mechanisms regulated by the IL-6R/JAK/STAT3 pathway, thereby sustaining tumor-promoting inflammation [90].

Metabolic and inflammatory signaling pathways also critically shape myeloid cell function. The nutrient-sensing kinase regulates macrophage and MDSC polarization in the TME [91]. Conversely, pro-inflammatory stimuli such as Toll-like receptors 7/8 agonists, IFN-γ, or TNF-α plus IL-6, can reprogram human Mo-MDSCs into M1-macrophages with antitumor activity via NF-κB and STAT4 signaling [92]. In esophageal cancer, elevated circulating MDSCs are associated with increased accumulation of M2-macrophages and a Th2-polarized immune profile, characterized by high IL-4, IL-13, IL-6, and GATA3, alongside reduced IFN-γ and IL-12 [93,94]. A similar pattern is observed in Hodgkin lymphoma, where the MDSC expansion correlates with the accumulation of pro-tumoral M2-macrophages [95].

Functional differences between myeloid subsets also emerge during tumor progression. In breast cancer models, MDSCs exhibit a more pro-inflammatory and pro-angiogenic profile and preferentially localize to hypoxic tumor regions, suggesting a role in early tumor development. As tumors progress, a shift toward TAMs is observed, with TAMs exerting stronger immunosuppressive effects and driving immune evasion [96]. At the molecular level, several regulators of myeloid cell function have been identified. The scavenger receptor MARCO is highly expressed in M2-TAMs and plays a key role in MDSC differentiation and immunosuppression. Its targeting reduces MDSC and TAM accumulation while enhancing CD8^+^ T-cells and NK cell responses [97]. Additionally, macrophages derived from Mo-MDSCs display a distinct immunosuppressive phenotype driven by persistent S100A9 expression and regulated by the transcription factor C/EBPβ. The presence of S100A9^+^ macrophages correlates with poor prognosis in patients with head and neck cancer [98].

Finally, in desmoplastic tumors such as cholangiocarcinoma (CCA), targeting TAMs alone induces a compensatory expansion of PMN-MDSCs, which sustains immune evasion by suppressing T-cell responses. Notably, a distinct ApoE^+^ PMN-MDSC subset emerges following TAM depletion and is also detectable in human CCA, underscoring the need for combinatorial strategies targeting multiple immunosuppressive myeloid populations [99].

## 5. Targeting PMN-MDSCs for Cancer Treatment

**Figure 3:**
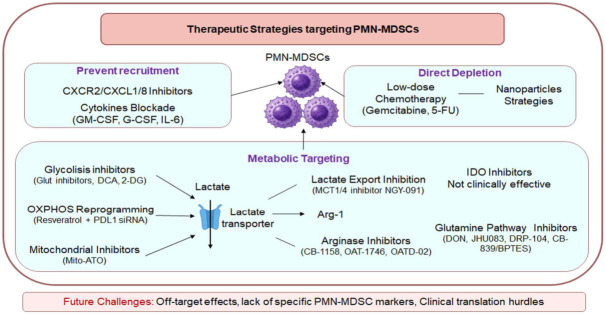
Targeting PMN-MDSC for cancer treatment. The main therapeutic approaches and challenges that are currently under investigation to counteract the immunosuppressive activity of PMN-MDSC. Strategies are grouped into three major categories: (i) prevention of PMN-MDSC recruitment to the TME through inhibition of chemokine signaling and cytokine blockade; (ii) direct depletion of PMN-MDSC using low-dose chemotherapy or nanoparticle-based approaches; and (iii) metabolic targeting, aimed at disrupting the metabolic pathways that sustain their survival and suppressive function, including glycolysis, oxidative phosphorylation, mitochondrial metabolism, lactate transport, arginase activity, glutamine metabolism, and IDO signaling.

A deeper understanding of the mechanisms through which MDSCs mediate immunosuppression is essential for the development of strategies aimed at restoring effective anti-tumor immunity (Figure 3).

Based on these acknowledgements, the major therapeutic strategies that can be applied to target PMN-MDSCs consist of preventing the recruitment or directly depleting them at the tumor site, limiting in this manner the interaction with other immune cells, and improving the local anti-tumor response. In addition, targeting the immunosuppressive mechanisms described above is a possible strategy. New insights from the literature focus on the importance of MDSC metabolism and the influence of TME-derived metabolites, although more studies in this field are needed. Several ongoing studies are investigating MDSC-targeting strategies in combination with other anti-cancer therapies to improve therapeutic efficacy.

### 5.1. Preventing PMN-MDSC Recruitment to Tumors

Tumors recruit PMN-MDSCs through chemokines and inflammatory cytokines. Blocking these pathways can reduce their accumulation in the TME. Targeting the CXCR2/CXCL1-CXCL8 axis is one of the most important pathways controlling PMN-MDSC migration [77]. Indeed, CXCR2 inhibitors have shown promise in preclinical models by improving T-cell infiltration and enhancing checkpoint blockade therapy, as well as targeting inflammatory tumor-derived cytokines (such as GM-CSF, G-CSF, IL-6) [100].

### 5.2. Depleting PMN-MDSCs Directly

Another therapeutic strategy involves the selective depletion of PMN-MDSCs, although the implementation of this approach remains challenging due to several biological and technical limitations. Indeed, some of the possible strategies, such as low doses of chemotherapeutic agents (e.g., gemcitabine, 5-fluorouracil), could affect the other immune cells. All-trans retinoic acid (ATRA), the active metabolite of vitamin A, has emerged as a key regulator of PMN-MDSC differentiation and immunosuppressive activity within the tumor microenvironment [101]. Several studies demonstrated that ATRA promotes the maturation of immature myeloid cells into differentiated non-suppressive populations, thereby reducing the accumulation and suppressive function of PMN-MDSCs through activation of the ERK1/2 pathway and increased glutathione synthesis, which neutralizes ROS-mediated immunosuppressive mechanisms [102,103,104]. Importantly, ATRA-mediated targeting of MDSCs has been associated with restoration of anti-tumor immune responses, including improved T-cell and NK cell activity. In preclinical and clinical settings, ATRA reduced circulating MDSC frequencies and enhanced responsiveness to immune checkpoint blockade, supporting its use as an adjuvant immunomodulatory strategy in cancer therapy [105].

Moreover, the use of antibodies targeting myeloid markers (e.g., nanoparticle-mediated drug delivery systems, etc.) is not yet feasible, since the common phenotype that PMN-MDSC shares with neutrophils, in fact, a unique antigen has not yet been characterized [8].

### 5.3. Targeting PMN-MDSC Metabolism

In the TME, the capability of MDSCs to rewire their metabolism and to promote the suppression of immune cells made it possible to investigate the potential targets of different pathways involved in their bioenergetic activity. Several strategies aim to disrupt glycolysis, since the PMN-MDSCs depend on it for their energetic capacity. For this reason, glucose depletion using agents such as 2-deoxyglucose (2-DG), inhibition of glucose transporters like GLUT3, and targeting metabolic enzymes such as pyruvate dehydrogenase with dichloroacetate (DCA) [106]. Additional approaches include metabolic reprogramming therapies, such as resveratrol, combined with PD-L1 siRNA, which shifts MDSCs from glycolysis back toward OXPHOS, reducing their suppressive activity and enhancing anti-tumor immunity [107]. Mitochondria-targeted atovaquone (Mito-ATO), a modified form of the antimalarial drug atovaquone, selectively impairs PMN-MDSC bioenergetics by inhibiting mitochondrial complex I and glycolytic pathways. This decreases PMN-MDSC survival, lowers Arg-1 and ROS production, and restores T-cell anti-tumor responses [108].

Enhanced glycolysis in MDSCs also leads to increased lactate production. Lactate is exported through monocarboxylate transporters, MCT1 and MCT4, to prevent intracellular acidification and sustain glycolytic activity. Extracellular lactate further activates MDSCs and strengthens their tumor-promoting and immunosuppressive functions [109]. Preclinical evidence suggests that dual inhibition of MCT1/4, including NGY-091, may attenuate lactate-driven immunosuppressive functions [110].

Although these metabolic-targeting strategies have shown promise in preclinical studies, their clinical application remains challenging because many normal immune cells in the tumor microenvironment also rely on glycolysis, raising concerns about off-target immune suppression. Also targeting the lipid metabolic pathways weakens PMN-MDSC suppressive activity, promotes anti-tumor immunity, and enhances the efficacy of immunotherapies, particularly immune checkpoint blockade [37].

Pharmacological inhibition of Arg-1 and, in some cases, Arg-2, using small molecules such as CB-1158, OAT-1746, and OATD-02, demonstrated restoration of T-cell anti-tumor response [24] and increased the arginine levels in TME, reduced the MDSC frequency [111]. Similarly, phosphodiesterase-5 (PDE5) inhibitors, such as sildenafil and tadalafil, downregulate Arg-1, iNOS, and inflammatory cytokine production, thereby attenuating MDSC-mediated immunosuppression and promoting T-cell infiltration and activation within the TME [112,113]. These immunomodulatory effects have also been translated into early clinical studies. In patients undergoing surgery for abdominal malignancies, perioperative sildenafil reduces the immunosuppressive activity of surgery-induced PMN-MDSCs and is associated with reduced postoperative recurrence, metastasis, and cancer-specific mortality [114,115]. Likewise, tadalafil treatment in patients with HNSCC and GBM significantly decreased both circulating and intratumoral MDSCs and Tregs, while increasing tumor antigen-specific CD8^+^ T-cell responses [116,117], supporting the clinical potential of PDE5 inhibition as an adjunct to cancer immunotherapy.

In contrast, molecules involved in tryptophan catabolism, such as IDO-1 inhibitors (e.g., INCB023843 and RY103), were developed to counteract tumor-induced immunosuppression, but they showed limited effects on MDSC functions and failed to improve clinical outcomes when combined with a PD-1 inhibitor in phase III clinical trials [118].

Targeting glutamine metabolism showed promising results; for example, 6-Diazo-5-oxo-L-norleucine (DON), a glutamine antagonist, showed anticancer properties. Due to the failure of DON in depleting MDSCs, some prodrugs of DON (e.g., JHU083, DRP-104, JHU395) that target the inhibition of glutamine pathways have emerged [119]. Pharmacological inhibition of glutaminase with CB-839 (telaglenastat), BPTES (bis-2-(5-phenylacetamido-1,2,4-thiadiazol-2-yl) ethyl sulfide), has been studied in different tumor types and in combination with other drugs; however, their direct impact on MDSCs depends on the tumor type, drug dosing, and compensatory pathways, which remain to be examined [95].

## 6. Concluding Remarks and Future Perspectives

MDSCs have evolved in scientific understanding from being viewed as simple immature myeloid populations to being recognized as sophisticated metabolic and regulatory hubs within the TME. Their extraordinary plasticity allows them to adapt to hostile conditions, effectively orchestrating a multi-layered immunosuppressive network through complex crosstalk with T-, B-, and NK-cells, as well as DCs and macrophages. In recent years, MDSC research has been reshaped by emerging technologies that enable more precise cell-state definition, tissue localization, and assessment of therapeutic relevance. Single-cell transcriptomics, multi-omics approaches, and spatial or multiplex imaging are beginning to resolve MDSC heterogeneity and assign specific functional states, linking them to suppressive niches and prognostic signatures [120,121,122]. These technologies are particularly valuable in the MDSC field, where universally validated unique markers are still lacking and where spatial context is essential to distinguish suppressive myeloid states from phenotypically similar populations, improving the possibility of identifying specific, solid, and valid markers for MDSC identification. Moreover, the future of MDSC-targeted therapy lies in moving beyond simple cellular depletion, which remains hindered by the lack of unique markers distinguishing PMN-MDSCs from mature neutrophils. Instead, the research focus is shifting toward functional reprogramming. By targeting the specific metabolic “engines” that drive their suppressive capacity, such as aerobic glycolysis, fatty acid oxidation, or the arginase and adenosine pathways, it may be possible to neutralize their inhibitory effects while preserving essential myeloid functions. Furthermore, overcoming resistance to current immunotherapies, particularly immune checkpoint inhibitors, will likely require combinatorial strategies. Ultimately, the identification of precise biomarkers and specific metabolic signatures will be crucial for personalizing these interventions and improving clinical outcomes in oncological patients.

## Data Availability

No new datasets were generated or analyzed in this narrative review. All cited data derive from previously published sources.

## Abbreviations

The following abbreviations are used in this manuscript:

MDSC	Myeloid-Derived Suppressor Cells
PMN-MDSC	Polymorphonuclear MDSC
Mo-MDSC	Monocytic MDSC
TME	Tumor Microenvironment
NK	Natural Killer
ROS	Reactive Oxygen Species
RNS	Reactive Nitrogen Species
NO	Nitric Oxide
DC	Dendritic Cells
TolDC	Tolerogenic Dendritic Cell
Treg	Regulatory T-Cells
B-reg	Regulatory B Cells
Arg-1	Arginase 1
IDO	Indoleamine 2,3-Dioxygenase
iNOS	Inducible Nitric Oxide Synthase
COX-2	Cyclooxygenase-2
PD-1	Programmed Cell Death Protein 1
PD-L1	Programmed Death-Ligand 1
FAO	Fatty Acid Oxidation
FATP2	Fatty Acid Transport Protein 2
PGE2	Prostaglandin E2
ATP	Adenosine Triphosphate
A2AR	Adenosine A2A Receptor
A2BR	Adenosine A2B Receptor
PBMC	Peripheral Blood Mononuclear Cells
PB	Peripheral Blood
CMP	Common Myeloid Progenitor
M-CSF	Macrophage Colony-Stimulating Factor
G-CSF	Granulocyte Colony-Stimulating Factor
GM-CSF	Granulocyte-Macrophage Colony-Stimulating Factor
IL	Interleukin
IFN-γ	Interferon Gamma
TNF-α	Tumor Necrosis Factor Alpha
JAK	Janus Kinase
STAT	Signal Transducer and Activator of Transcription
TCR	T-Cell Receptor
MHC	Major Histocompatibility Complex
APC	Antigen-Presenting Cells
LOX-1	Lectin-Type Oxidized LDL Receptor 1
LDL	Low-Density Lipoprotein
MPO	Myeloperoxidase
TAM	Tumor-Associated Macrophages
MARCO	Macrophage Receptor with Collagenous Structure
NF-κB	Nuclear Factor Kappa B
C/EBPβ	CCAAT/Enhancer-Binding Protein Beta
HNSCC	Head and Neck Squamous Cell Carcinoma
GBM	Glioblastoma
CCA	Cholangiocarcinoma
ATRA	All-Trans Retinoic Acid
ERK1/2	Extracellular Signal-Regulated Kinases 1/2
GLUT3	Glucose Transporter 3
2-DG	2-Deoxyglucose
DCA	Dichloroacetate
OXPHOS	Oxidative Phosphorylation
Mito-ATO	Mitochondria-Targeted Atovaquone
MCT1/4	Monocarboxylate Transporters 1/4
CAR T-cells	Chimeric Antigen Receptor T-Cells
TIGIT	T-cell immunoreceptor with Ig and ITIM domains
Ig	Immunoglobulin
